# The Paradox of Citizenship Cost: Examining a Longitudinal Indirect Effect of Altruistic Citizenship Behavior on Work–Family Conflict Through Coworker Support

**DOI:** 10.3389/fpsyg.2021.661715

**Published:** 2021-05-07

**Authors:** Sajid Haider, Carmen De-Pablos-Heredero, Monica De-Pablos-Heredero

**Affiliations:** ^1^Department of Management Sciences, COMSATS University Islamabad, Vehari, Pakistan; ^2^Department of Business Economics, Rey Juan Carlos University, Móstoles, Spain; ^3^Esic Business & Marketing School, Madrid, Spain

**Keywords:** altruistic citizenship behavior, citizenship cost, paradox, coworker support, work-family conflict

## Abstract

The objective of this study was to address the paradox of citizenship cost by hypothesizing an indirect rather than a direct effect of altruistic citizenship behavior (ACB) on employee work–family conflict (WFC) through coworker support (CWS). Data were gathered in a three-wave longitudinal survey of employees from private commercial banks (*N* = 318). A multiple linear autoregressive longitudinal mediation model was analyzed with partial least squares structural equation modeling (PLS-SEM). The results indicate that rather than directly, ACB affects indirectly employee WFC through CWS. This indirect effect is negative, which reflects that the costs of citizenship behavior are paradoxical. The present study contributes to the ongoing debate on the positive and negative outcomes of employee citizenship behavior by providing empirical evidence on the beneficial rather than harmful effect of performing such behavior. For organizational managers, promoting a culture of CWS by encouraging altruistic behaviors can be a most viable strategy to reduce WFC among their employees. The study discusses its limitations and provides future research directions.

## Introduction

Despite a large body of research supporting positive outcomes of organizational citizenship behavior (OCB), researchers’ interest in addressing the personal costs associated with exhibiting such behaviors is growing (Van Dyne and Ellis, 2004; Bolino and Turnley, 2005; Vigoda-Gadot, 2006; Bergeron, 2007; Halbesleben et al., 2009; Bolino et al., 2013, 2015; Deery et al., 2017). The proponents of this line of research posit that advances in OCB research require attention to its dark side (Bolino and Grant, 2016) and insist on considering a “balanced perspective” by recognizing also the negative outcomes of OCB (Lennard and Van Dyne, 2016).

Following the dark side’s reasoning, previous research has examined a number of negative consequences of OCB. For example, Bolino and Turnley (2005) found that “higher levels of individual initiative (a specific type of OCB) are related to higher levels of role overload, job stress, and work–family conflict” (p. 744). Bolino et al. (2015) noticed that engaging in OCB produced more citizenship fatigue when employees were faced with low organizational support and leader–member exchange, and high citizenship pressure. Recently, Deery et al. (2017) found that altruism and conscientiousness (two dimensions of OCB) were positively associated with emotional exhaustion and work–family conflict (WFC) for employees with high in-role performance.

Among other employee level “costs” of OCB, the dark side researchers’ greater interest has been observed in WFC, which is a widespread phenomenon in work life, and has been studied in diverse disciplines worldwide (French et al., 2018; Ahmad and Islam, 2019; Masuda et al., 2019). Researchers have recognized it as “a prominent societal concern” (French et al., 2018). Work–family conflict refers to “a form of inter-role conflict in which the role pressures from the work and family domains are mutually incompatible in some respect” (Greenhaus and Beutell, 1985, p. 77). It reflects “a situation in which work-related stress and family responsibilities interfere with each other” (Islam et al., 2020, p. 404). The proponents of dark side argue that OCBs are “likely to contribute to higher levels of work interference with family” (Halbesleben et al., 2009, p. 1453).

The findings of dark side studies are based on strong theoretical logic and sophisticated analyses of data. However, the estimated causality between OCB and “personal costs” may not represent the true effect of OCB because other logical influences (i.e., mediating or suppressing effects) might be affecting the results otherwise. As the bivariate effect between endogenous and exogenous variable “obscures the complexity of the causal relations between these variables” (Shrout and Bolger, 2002, p. 431), previous research on the direct effect of OCB on an employee’s personal costs (such as WFC) might have found misleading results. It can be true, specifically, in longitudinal studies where theoretically interesting relationships may become empirically weak over time (Shrout and Bolger, 2002). We argue that without considering the possible intervening mechanisms over time, the validity of true cause–effect relationship may remain ambiguous.

Evolutionary biology’s point that human species in all cultures meet the preconditions for exhibiting altruism (a specific form of OCB) (Gouldner, 1960; Trivers, 1971) suggests that employees performing altruistic behaviors toward their colleagues are highly likely to receive these behaviors in the form of coworkers’ support in future events (Halbesleben and Wheeler, 2015). Altruism or “altruistic behavior can be defined as a behavior that benefits another organism, not closely related, while being apparently detrimental to the organism performing the behavior” (Trivers, 1971, p. 35). Coworker support (CWS) refers to the “amount of instrumental aid, emotional concern, informational, and/or appraisal functions from peers or coworkers” (Michel et al., 2011, p. 693). It is well recognized in organizational behavior literature that CWS is negatively associated with WFC (Thomas and Ganster, 1995; Jansen et al., 2003; Ford et al., 2007; Dolcos and Daley, 2009; Mesmer-Magnus and Viswesvaran, 2009; Michel et al., 2010, 2011; Norling and Chopik, 2020).

Under certain conditions in future interactions, altruistic behaviors benefit the organism who performed altruism earlier (Trivers, 1971; Lozada et al., 2011). Employees who invest time and energy to perform altruistic behaviors toward coworkers may hope “that their investment will be duly reciprocated by those employees in the future” (Halbesleben and Wheeler, 2015, p. 610). Existing literature confirms that altruistic behaviors are “likely to be antecedents of the receipt of social support” (Bowling et al., 2004, p. 347) and “any type of social support provided by one person in the work setting could be reciprocated with the same or different types of social support” (Bowling et al., 2005). Given that altruistic behaviors are reciprocated in the form of CWS, it can be stated that performing these behaviors is likely to reduce over time the negative employee outcomes such as WFC.

Based on the norms of reciprocity (Gouldner, 1960) or reciprocity theory (Bowling et al., 2004), this study posits that employees who perform altruistic citizenship behavior (ACB) are less likely to fall victim of WFC as they are highly likely to receive, over time, the CWS that is negatively associated with WFC. As reciprocity is an evolutionary process where cooperation is expected in response to cooperation (Rosas, 2008), the intervening role of CWS is quite justifiable when the effect of altruistic behavior on WFC is examined. Given that altruistic behaviors are reciprocated, support from coworkers is likely to intervene to reduce WFC (Dolcos and Daley, 2009). In order to develop argument for the intervening role of CWS, this study has used insights mainly from reciprocity theory (Bowling et al., 2004), social support theory (Shumaker and Brownell, 1984), social support resource theory (Hobfoll et al., 1990), and conservation of resources (COR) theory (Hobfoll et al., 1989). Moreover, the argument has been strengthened by integrating these theories’ insights with role theory (Kahn et al., 1964; Katz and Kahn, 1978), resource drain theory (Edwards and Rothbard, 2000; Morris and Madsen, 2007), and equity theory (Adams et al., 1976).

Based on these theoretical insights, this study posits that altruistic behaviors help employees to conserve personal resources in the form of social support, which, in turn, helps reduce WFC over time. It leads us to believe that the relationship between altruistic behavior and WFC is a multivariate rather than a bivariate phenomenon. So, this study assumes that the indirect effect of altruistic behavior on WFC through CWS reflects a true causal relationship, while the direct effect is meaningless when the intervening mechanisms (i.e., CWS in this study) are held constant (MacKinnon et al., 2000; Shrout and Bolger, 2002). Empirical analysis of the abovementioned phenomenon may render such results that support indirect rather than a direct effect. Unfortunately, previous research lacks empirical evidence on such phenomena.

This study seeks to fill this gap by theorizing and longitudinally examining the indirect effect of employee altruism or ACB (Wagner and Rush, 2000) on WFC through CWS. So, the focus of this study is indirect effect rather than a direct effect. It is important because the indirect effects, rather than direct effects, “can be of theoretical and practical importance” (Rucker et al., 2011, p. 368). Moreover, despite many theoretical elaborations of the effects of altruism, empirical research in this area is scarce (Brase, 2017). Our research is likely to address the issue of this scarcity.

This study contributes to the ongoing debate on positive and negative outcomes of citizenship behavior by providing empirical evidence on beneficial effect of such behavior in an indirect longitudinal model. Previous research on the costs of citizenship behavior has ignored that altruistic behaviors may not sustain if not reciprocated because these behaviors cannot evolve if the donors mostly bear the net cost (Sober, 1992). As people are sensitive to costs and benefits of exhibiting and reciprocating altruistic behaviors (Nowak and Sigmund, 2005), the citizenship costs cannot sustain when reciprocity factor such as CWS is considered over time. If the basic rule in the evolutionary norms of reciprocity is “cooperation in response to cooperation, defection in response to defection” (Rosas, 2008, p. 557), one should accept that the effects of altruistic behaviors evolve over time in the form of CWS, which helps reduce WFC. It suggests that the bivariate direct positive association of citizenship behavior with WFC in previous cross-sectional studies is paradoxical. This study contributes to organizational psychology literature by addressing this paradox.

The rest of the work is organized as follows. The next section, theory and hypotheses, is about this study’s theoretical framework where hypotheses have been developed by using theoretical insights from existing literature. The section next to “Theory and Hypotheses” is “Materials and Methods”. This is followed by section “Results”, and the final section is about discussion on this study’s findings, practical and theoretical implications, and limitations and future research.

## Theory and Hypotheses

Previous research has provided strong theoretical argument and robust findings about the positive relationship between altruistic OCB and WFC (Bolino and Turnley, 2005; Deery et al., 2017; Liu et al., 2017). However, the bivariate effect of altruistic OCB on WFC conceals the complexity of relationship between these two phenomena. Little work has investigated the processes that may differently affect the relationship between ACB and WFC. So, this study is focused on developing argument for indirect negative relationship between ACB and WFC. In light of previous research (Rucker et al., 2011), we argue that indirect effect, rather than a direct effect, provides a more concise assessment of the relationship between ACB and WFC.

**FIGURE 1:**
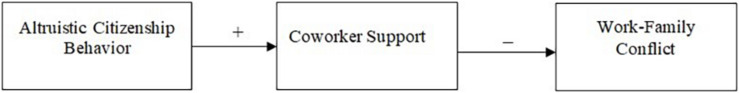
Theoretical model.

Figure 1 shows this study’s theoretical model where ACB is related to employee WFC indirectly through CWS. The indirect process is partially positive (from ACB to CWS) and partially negative (from CWS to WFC). This model is based on the assumption that altruistic behavior is less likely to lead to WFC in the presence of CWS. Based on norms of reciprocity or reciprocity theory, this study argues that altruistic acts are normally reciprocated in the form of support from those who received support/help (or altruism) earlier (Hamilton, 1964; Hoffman, 1981; Cosmides and Tooby, 1992; McCullough et al., 2008; Lozada et al., 2011). Given that altruistic employees are highly likely to receive CWS, this study claims that such employees are less likely to experience WFC because CWS helps reduce WFC (Dolcos and Daley, 2009). In other words, this study claims that altruistic employees would certainly succeed in reducing their WFC as they receive CWS in response to their altruistic behavior.

### Altruism and Coworker Support

Insights from norms of reciprocity (Gouldner, 1960) or reciprocity theory (Bowling et al., 2004) provide strong argument for the existence of altruistic behaviors among human beings (Hoffman, 1981; Lozada et al., 2011). Human vulnerability to the persistence of endangerments in their environment makes them acknowledge the benefits of performing helping behavior (or altruism) in their own self-interest, specifically when they believe that the whole population is susceptible to such vulnerabilities (Hoffman, 1981; Lozada et al., 2011). Given that altruism is necessary for survival, the evolution of human organism should not be expected without selecting those physical and genetic characteristics that help perform altruism. Therefore, altruism is a strong human trait that warrants survival as it helps adaptation during natural selection (Hamilton, 1964; Cosmides and Tooby, 1992; McCullough et al., 2008).

Though the preliminary work on Darwinian thoughts of “survival of the fittest” promoted human beings as egoistic and self-preserving, later evidence suggested that humans have subsisted the ever-prevailing unfavorable conditions by forming groups, and consequently, the notion of “cooperative social existence” took ground within the Darwinian model (Hoffman, 1978). Hence, besides being egoistic, human beings own altruistic structures that produce helping behaviors and stimulate interpersonal facilitation (Hoffman, 1981; Hurtz and Donovan, 2000; Judge et al., 2009).

Previous experimental research on humans’ and chimpanzees’ reciprocity to altruistic behaviors shows that altruism is normally positively reciprocated for both food and non-food items or services (Bethell et al., 2000; Mitani and Watts, 2001; Nakamura and Itoh, 2001; Slocombe and Newton-Fisher, 2005; Hockings et al., 2007; Yamamoto and Tanaka, 2009). Meta-analytic work on the manifestation and reciprocity of altruism in primates, including humans, has reported strong positive correlation between these two phenomena. For example, Jaeggi and Gurven (2013) found that the correlation between the occurrence of altruistic act of food sharing and its reciprocation in humans and other primates ranged from 0.20 to 0.48. Schino and Aureli’s (2008) meta-analysis reported a high weighted correlation (0.47) between the given and received grooming among primates.

It follows from the biological theory and evidence that some behavioral mechanisms might be necessary for performing altruism (Hoffman, 1981). Human altruism with a “strong cognitive component” is “supported by different psychological mechanisms” (Warneken and Tomasello, 2009, p. 457). Insights from evolutionary psychology inform that mutation and selection processes produce not only the physical but also the psychological traits such as conscientiousness and agreeableness, which aid survival by fostering prudence and cooperation within and between groups (Judge et al., 2009). So, natural selection supports in human beings a complementary but complex psychological system that regulates their likelihood to perform altruistic behaviors and guides how to respond others’ altruistic orientations (Trivers, 1971).

An important aspect of this complex system is its sensitivity to cost/benefit ratios in deciding whether to respond to an altruistic act and how much to reciprocate (Trivers, 1971; Wilson, 1990; Sober, 1992; Nowak and Sigmund, 2005). People receiving altruism are likely to reciprocate to the donor of altruistic act with the same or greater benefit because altruistic behaviors cannot evolve if the donors mostly bear the net cost (Sober, 1992). So, the existence of altruistic acts in social or organizational settings is not without the fact that such acts are reciprocated. Though individual difference and problems of cheaters exist, the fact that altruistic acts are reciprocated is well recognized in evolutionary biology, evolutionary psychology, social psychology, and anthropology. This is because the reciprocity of altruistic acts evolves those cognitive powers in humans which perpetuate reciprocal gain spirals (Trivers, 2006; Halbesleben and Wheeler, 2015).

The social systems, either in a society or in an organization, sustain stability based on the norms of reciprocity (Gouldner, 1960). In a broader sense, the norm of reciprocity refers to the idea that the recipient of a benefit in a social system feels the obligation of returning that benefit to the benefactor (Robinson et al., 1994). People tend to avoid being indebted or over-benefiting from the social support they receive from others (Uehara, 1995). Empirical investigations into the evolution of finite populations suggest that natural selection favors reciprocity to become a norm or a “stable strategy” in social systems because everyone needs to behave cooperatively (André and Day, 2007; Warneken and Tomasello, 2009).

Based on Gouldner’s (1960) principle of reciprocity, previous empirical research in work and non-work settings has recognized that the donors of altruistic or helping behaviors receive social support from the recipients of those behaviors. For example, Acitelli and Antonucci (1994) found that the correlations between social support and reciprocal support were 0.77 and 0.81 for husbands and wives, respectively. Likewise, Jou and Fukada (2002) found this correlation as 0.71 among college students. In work settings, Bowling et al. (2004) argued that employees who behave altruistically toward their coworkers are highly likely to receive support from them. These authors found a correlation of 0.17 between individual OCB and support from coworkers.

Besides the abovementioned explanations of altruism and its reciprocity, many theories support the assumption that altruistic behaviors are reciprocated. For example, Falk and Fischbacher’s (2006) reciprocity theory takes humans as reciprocal; “people reward kind and punish unkind actions” (p. 309). According to equity theory (Adams et al., 1976; Walster et al., 1978), individuals tend to avoid guilt by putting extra effort when they perceive themselves over-rewarded in a relationship. Individuals who receive altruism from their coworkers are highly likely to reciprocate that help or support to maintain equity, and thus the consistency of altruistic behaviors is maintained across coworkers (Bommer et al., 2003; Bowling et al., 2004). COR theory (Hobfoll et al., 1989) assumes that people invest their current resources to clinch personal resources in future (Halbesleben and Wheeler, 2015). Based on this assumption, Halbesleben and Wheeler (2015) hypothesized that “Coworker investment of resources in an employee (in the form of OCBs) will increase that employee’s perception of available resources (in the form of social support)” (p. 1632). It follows from this assumption that altruistic employees are highly likely to receive support from their coworkers, when they need. The above discussion leads us to the following hypothesis.

Hypothesis 1: Altruistic citizenship behavior is positively associated with coworker support.

### The Intervening Role of Coworker Support

Given that altruistic behavior leads to CWS, one can argue about the negative effect of altruism on WFC through CWS. Altruistic behavior allows employees to reduce their WFC mainly because it enables them to receive CWS (Bowling et al., 2004). However, to explain why altruistic behavior may negatively influence WFC through CWS, one needs to justify how CWS reduces WFC. Empirical evidence from existing research indicates that employees’ WFCs increase in an unsupportive work environment and decrease when the organization, supervisors, and coworkers are supportive (Greenhaus and Beutell, 1985; Frone et al., 1997; Michel et al., 2011). As a social resource at workplace, CWS helps to integrate work and family demands, and reduces WFC in both public and private sector employees (Brough and Pears, 2004; Thompson and Prottas, 2006; Dolcos and Daley, 2009). In other words, coworkers play a vital role in reducing employees’ WFC as they provide social support and enhance wellbeing (Michel et al., 2011; Halbesleben and Wheeler, 2015; Haider et al., 2018). Existing literature suggests that CWS not only reduces WFC but also helps “in alleviating the detrimental impact of work–family conflict on exhaustion” (Karatepe, 2009, p. 836). Moreover, CWS reduces WFC by moderating the effect of workload on emotional exhaustion (Pluut et al., 2018).

In existing literature, two major theoretical perspectives have been used to delineate the relationship between workplace social support and WFC. First, the role theory perspective predicates that “life domains, such as work and family, entail multiple roles where demands are placed on the individual, often resulting in conflict” (Michel et al., 2011, p. 92). However, social support resource theory (Hobfoll et al., 1990) suggests that people strive “to maintain social support both to meet their needs to preserve particular resources and in order to protect and maintain their identity” (Hobfoll et al., 1990, p. 467). It implies that people maintain a certain level of social support either by behaving altruistically or by other means. Given that people maintain social support, workplace social support may decrease an employee’s WFC because the “supportive members of a person’s role set(s) may directly reduce certain role pressures” (Greenhaus and Beutell, 1985, p. 86). It is true because people seek social support when their role involvement tends to create WFC (Wheaton, 1985). So, role burden or pressure is less likely to produce WFC because people maintain a certain level of social support by supporting or helping others. Based on role theory, Michel et al. (2011) hypothesized that support at workplace (including support from coworkers) is negatively associated with WFC, and found support for this hypothesis. They further argued that “both role theory and resource drain theory imply an inverse relationship between social support and work–family conflict” (p. 698).

Second, the resource perspective posits that social support reduces WFC because it improves an individual’s resource portfolio (Thomas and Ganster, 1995; Ford et al., 2007; Michel et al., 2010). Though resource drain theory assumes that an individual’s unique resources (such as energy and time) are limited in supply, and may not be available for family domain if spent in work domain (Valcour, 2007; Deery et al., 2017), Hobfoll et al.’s (1989) COR theory suggests that resource drain is reversible or even restored through support from coworkers (Brotheridge and Lee, 2005).

It follows that employees preserve CWS as a conserved resource when they want to avoid WFC. The resources conserved in the form of social support reduce attention, energy, and time required to accomplish work roles, and thus may reduce WFC by adding resources in family domain (Michel et al., 2011). The reasoning behind the pervasiveness of resource conservation rather than resource drain over time can be found in social support theory (Shumaker and Brownell, 1984). Social support theory defines social support as “an exchange of resources between at least two individuals perceived by the provider or the recipient to be intended to enhance the wellbeing of the recipient” (Shumaker and Brownell, 1984, p. 11). This theory asserts that “there are potential costs and benefits associated with the exchange for both participants” (Shumaker and Brownell, 1984, p. 13). Social support perspective asserts that prosocial or altruistic behaviors and norms of reciprocity guide how the benefits and costs are assessed.

The concept of reciprocity hinges upon mutual obligation (Cobb, 1976), which demands support in response to support (Greenberg, 1980). It implies that existence of social support reflects the continuity of supportive relationships between the recipient and provider (Shumaker and Brownell, 1984). The fact that support from coworker reduces WFC indicates that the employee in question has conserved this support as a response to helping behavior performed earlier. The literature on prosocial behavior suggests that the provider’s decision to perform helping behavior is influenced by the recipient’s various traits including social skills and values about giving help (Raven and Rubin, 1983; Shumaker and Brownell, 1984). It implies that the receipts of altruistic behaviors are likely to be the people who conserve the similar help or support rather than draining the provider’s resources such as time and energy.

As a key dimension of OCB, altruism implies helping coworkers “when they have heavy workloads or listening to their problems” (Bowling et al., 2004, p. 340), and thus maintains consistency of helping behaviors among coworkers (Bommer et al., 2003). Equity theory suggests that the recipients of altruistic behavior are highly likely to return that altruism to the donor in future events. It follows from this idea that the consistency of altruism is highly likely across coworkers, which means that employees not receiving help (from those to whom they provide help) are more likely to avoid equity tension by reversing their willingness to continue engaging in altruism. Based on equity theory and the principle of reciprocity, it further follows that a burdensome situation of exhibiting altruism is less likely to occur, especially when it creates WFC for the donor. In some situations, people may continue less costly (or zero cost) altruism even when they expect little reciprocity, but the continuity in voluntarily performing a costly behavior makes little sense. The proponents of the dark side of OCB seem to ignore the fact that neither human biology and psychology nor the principles of reciprocity and equity allow the sustainability of vexatious altruistic behaviors. If there is altruism, it is because the recipients are reciprocating it, and thus the consistency of altruistic behaviors is maintained. So, an employee’s altruistic behavior may reduce that employee’s WFC as it increases the receipt of support from coworkers, which is negatively associated with WFC. Based on this, we hypothesized the following:

Hypothesis 2a: Coworker support is negatively associated with work–family conflict.
Hypothesis 2b: There is a negative indirect effect of altruistic citizenship behavior on work–family conflict through coworker support.

## Materials and Methods

### Sample and Procedures

Data were collected in three waves by using a 6-month time lag. The study subjects were full-time employees of private commercial banks in south Punjab (Pakistan). Banks provide suitable settings for research surveys as they possess well-established organizational structures and qualified employees (Haider et al., 2019a). An informed consent from the survey participants and approval from the Ethical Committee for Scientific Research were obtained before data collection. This study used multiple data sources by obtaining supervisor ratings and employee self-ratings.

Sample size was determined by using insights from Cohen (1992) as described in Hair et al. (2014). These insights recommend a sample of 205 for detecting an *R*^2^ value of 0.1 (with 1% probability of error), and obtaining statistical power of 80%, when a maximum of five arrowheads point at an endogenous variable in a model (as is the case in this study). Taking into account the need for three-wave panel data for performing longitudinal analysis, the target sample was much larger (more than three times) than the recommended sample size because the participants “who complete the first wave of the survey fail to participate in subsequent waves” (Hillygus and Snell, 2015, p. 1). This study used simple random sampling technique to select a sample of 680 from 1452 employees working in 158 bank branches of private commercial banks in the target districts of south Punjab. As a probability sampling technique, simple random sampling reduces biases in selecting a sample as it gives equal chance of selection to each member of the population (Haider et al., 2019b). Moreover, it allows a researcher to posit “how confident he/she is that the research results reflect the situation in the underlying population” (Reynolds et al., 2003, p. 88).

In the first wave, 680 randomly selected employees and their respective supervisors were provided with paper-based survey questionnaires. Each employee was assigned a distinct code so that the responses could be matched with respect to supervisors and data waves. All study variables were measured in all three waves. However, the data were used as was required by the procedures of data analysis. The first wave survey was closed with 511 supervisor-subordinate matched usable responses.

After 6 months of the completion of the first-wave survey, the questionnaires were prepared for 511 subjects who completed the survey in the previous wave. However, two of these 511 employees had left jobs, and three were on long-term leave. So, the second-wave survey was conducted on 506 subjects. The second wave obtained 429 usable responses. The third-wave survey was administered after 6 months from the completion of the second-wave survey. The third wave surveyed 426 out of 429 subjects as three employees were on long-term leave. A total of 318 (47% from first to third wave) supervisor–subordinate matched usable responses were received for the same employees in all three waves.

### Demographics of Study Sample

The final sample comprised 189 (60%) male and 129 (40%) female employees, who were rated by 45 supervisors (seven female). The mean age and experience of employees were 27.5 and 5.9 years, respectively. Table 1 shows the descriptive statistics of the study sample. There are two notable things with respect to the study sample as shown in Table 1. First, there is a gender disparity, i.e., 59% male and 41% female. This disparity, however, is a national phenomenon (Anjum et al., 2019) and cannot be avoided in many cases. Second, the sample is relatively young. According to Pakistan Bureau of Statistics (2015) and Pakistan Economic Survey (2017), Pakistan has a young labor force. So, it is normal to have a young sample.

**TABLE 1:** Descriptive statistics.

**Description**	**Classification**	**Frequency**	**Percentage**
Gender	Male	189	59
	Female	129	41
Age	21–30	235	74
	31–40	59	19
	41–50	21	7
	Above 50	3	1
Experience	2–10 years	270	85
	11–20 years	48	15
Qualification	Intermediate	92	29
	Graduation	197	62
	Masters	29	9

### Measures

#### Altruistic Citizenship Behavior

Altruistic citizenship behavior was measured by using a five-item altruism scale used in Podsakoff et al. (1990).

#### Work–Family Conflict

Netemeyer et al.’s (1996) five-item scale was used to measure WFC.

#### Coworker Support

Coworker support was measured with a five-item instrumental support scale used in Ducharme and Martin (2000).

These scales were validated by using quality criteria in partial least squares structural equation modeling (PLS-SEM). The validation procedures have been explained under the section “Evaluation of Measurement Model.” Employee self-ratings were obtained for WFC and CWS scales. For ACB, employees were rated by their respective supervisors. All the ratings were obtained at a five-point Likert scale ranging from 1 (strongly disagree) to 5 (strongly agree).

### Control Variables

Existing literature informs that WFC is affected by employees’ gender (Cinamon, 2006) and tenure (Karatepe, 2009). So, this study controlled for the participant employees’ gender (0, “male”; 1, “female”) and tenure (in years).

### Analytical Approach

This study used a three-wave autoregressive time-lagged model for examining causal relationships in a mediation model (Cole and Maxwell, 2003). This autoregressive model was tested by using partial least squares structural equation modeling (PLS-SEM) in SmartPLS software, version 3.2.7 (Ringle et al., 2005). PLS-SEM works with component-based estimation procedures by using iterative algorithms of least squares regressions (Hair et al., 2014; Haider et al., 2020). In light of Hair et al. (2017) and Mishra et al. (2016), Haider et al. (2020) explained that PLS-SEM tests hypotheses “based on resampling method—bootstrapping. … It is considered as an efficient tool for data analysis because it allows simultaneous estimation of item loadings and path coefficients, minimizes biases, and reduces measurement error” (p. 6). PLS-SEM is advantageous over covariance-based SEM (CB-SEM) because it allows good results from small datasets and does not require normal distribution in data. PLS path models operate through measurement and structural models for data validity and hypothesis testing, respectively.

## Results

### Evaluation of Measurement Model

Table 2 exhibits factor loadings of each individual item and Cronbach’s alpha (α), composite reliability (CR), and average variance extracted (AVE) for each reflective latent variable used in this study. These values are used to measure internal consistency reliability, convergent validity, and discriminant validity of study variables. The values of alpha (α) and CR above 0.70 indicate that a construct is internally consistent, which means that all items of that construct are equally reliable. This study’s all variables are internally consistent as alpha and CR values are above 0.70 (Table 2). In Table 2, there is a notable difference between Cronbach alpha (α) and CR values for Time 2 altruistic citizenship behavior (T2-ACB). However, existing research indicates that the value of Cronbach alpha can be less than the value of CR because alpha is a lower bound estimate of reliability and underestimates internal consistency (Raykov, 2001; Peterson and Kim, 2013; Haider et al., 2018).

**TABLE 2:** Evaluation of measurement model.

**Constructs**	**Indicators**	**λ^a^**	**α^b^**	**CR^c^**	**AVE^d^**
Time 1 work–family conflict (T1-WFC)	T1-WFC1	0.83	0.90	0.93	0.78
	T1-WFC2	0.91			
	T1-WFC3	0.91			
	T1-WFC4	0.87			
Time 2 work–family conflict (T2-WFC)	T2-WFC1	0.82	0.87	0.91	0.72
	T2-WFC2	0.88			
	T2-WFC3	0.90			
	T2-WFC4	0.79			
Time 3 work–family conflict (T3-WFC)	T3-WFC1	0.72	0.89	0.92	0.74
	T3-WFC2	0.90			
	T3-WFC3	0.92			
	T3-WFC4	0.89			
Time 1 altruistic citizenship behavior (T1-ACB)	T1-ACB2	0.93	0.92	0.94	0.83
	T1-ACB3	0.95			
	T1-ACB4	0.95			
	T1-ACB5	0.77			
Time 2 altruistic citizenship behavior (T2-ACB)	T2-ACB2	0.66	0.71	0.79	0.51
	T2-ACB3	0.76			
	T2-ACB4	0.80			
	T2-ACB5	0.64			
Time 1 coworker support (T1-CWS)	T1-CWS1	0.72	0.91	0.92	0.86
	T1-CWS2	0.95			
	T1-CWS3	0.96			
	T1-CWS4	0.95			
	T1-CWS5	0.94			
Time 2 coworker support (T2-CWS)	T2-CWS1	0.63	0.89	0.90	0.80
	T2-CWS2	0.97			
	T2-CWS3	0.97			
	T2-CWS4	0.95			
	T2-CWS5	0.88			

*^*a*^Factor/outer loadings. ^*b*^Cronbach’s alpha. ^*c*^CR, composite reliability. ^*d*^AVE, average variance extracted.*

Each individual item’s factor loading above or equal to 0.70 and AVE value above or equal to 0.50 are the quality criteria for establishing convergent validity, which denotes “the extent to which a measure correlates positively with alternative measures of the same construct” (Hair et al., 2014, p. 102). However, in case of factor loadings, items with loadings between 0.40 and 0.70 may remain with the construct under some conditions. According to Hair et al. (2014), “indicators with outer loadings between 0.40 and 0.70 should be considered for removal only if the deletion leads to an increase in composite reliability and AVE above the suggested threshold value” (p. 107). The items having factor loadings below 0.40 must be deleted from the construct (Hair et al., 2014). The AVE values and factor loadings in Table 2 meet the above quality criteria except one item of ACB (ACB1) in both waves 1 and 2. This item (T1-ACB1 and T2-ACB1) was deleted from the construct because its factor loadings was lower than 0.40 in both waves (Hair et al., 2014). Deleting an item from a reflective construct does not change its meaning if the construct level reliability criteria are met (Jarvis et al., 2003). The outer loadings for T2-ACB2, T2-ACB4, and T2-CWS1 were slightly lower than 0.70. The deletion of these items did not increase the CR and AVE of the related construct. So, the items were retained with their respective constructs.

Discriminant validity was established to assure that each individual construct has its distinct position in relation to other constructs. This study established discriminant validity by using the latest technique, heterotrait–monotrait (HTMT) ratios of correlations, as it has advantage over the traditional Fornell and Larcker (1981) criterion and cross-loadings method (Henseler et al., 2015). Henseler et al. (2015) defined HTMT ratio as “the average of the heterotrait-heteromethod correlations (i.e., the correlations of indicators across constructs measuring different phenomena), relative to the average of the monotrait heteromethod correlations (i.e., the correlations of indicators within the same construct)” (p. 121). According to the strictest criterion (conservative approach) described in Henseler et al. (2015), the value of HTMT ratio between two constructs must be less than 0.85 (HTMT0.85). In Table 3, all the HTMT ratios less than 0.85 indicate that discriminant validity has been established between the study constructs.

**TABLE 3:** Heterotrait–monotrait (HTMT) ratios of correlations.

	**Gender**	**Tenure**	**T1_ACB**	**T1_CWS**	**T1_WFC**	**T2_ACB**	**T2_CWS**	**T2_WFC**	**T3_WFC**
Gender									
Tenure	0.077								
T1_ACB	0.022	0.236							
T1_CWS	0.038	0.367	0.109						
T1_WFC	0.195	0.038	0.044	0.147					
T2_ACB	0.039	0.194	0.173	0.164	0.172				
T2_CWS	0.147	0.107	0.317	0.226	0.038	0.205			
T2_WFC	0.031	0.529	0.471	0.105	0.290	0.459	0.194		
T3_WFC	0.107	0.349	0.175	0.192	0.087	0.148	0.105	0.202	

*T1, time 1; T2, time 2; T3, time 3; ACB, altruistic citizenship behavior; WFC, work–family conflict; CWS, coworker support.*

### Evaluation of Structural Model

Evaluation of structural model is concerned with testing the magnitude and significance of hypothesized relationships. Before hypotheses testing in a reflective measurement model, each set of predictor variables is assessed for collinearity issues (Hair et al., 2014). Collinearity is normally assessed by using variance inflation factor (VIF). A VIF value of less than five indicates absence of collinearity between each set of predictors. As shown in Table 4, there is no collinearity issue in our model.

**TABLE 4:** Collinearity assessment (VIF values).

	**T2_ACB**	**T2_CWS**	**T2_WFC**	**T3_WFC**
Gender				1.040
Tenure				1.319
T1_ACB	1.000	1.004	1.005	1.330
T1_CWS		1.004	1.023	
T1_WFC			1.020	
T2_ACB				1.172
T2_CWS				1.165
T2_WFC				1.652
T3_WFC				

*T1, time 1; T2, time 2; T3, time 3; ACB, altruistic citizenship behavior; WFC, work–family conflict; CWS, coworker support.*

### Hypotheses Testing

**FIGURE 2:**
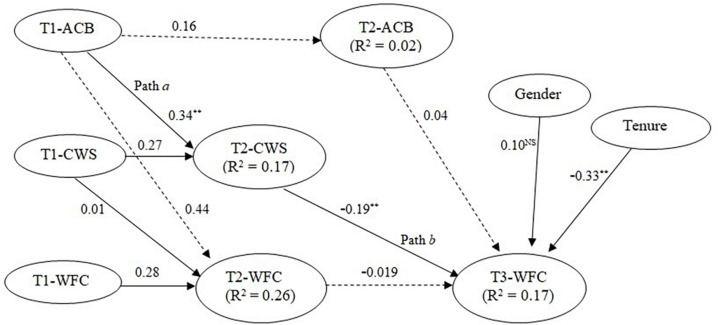
Estimated longitudinal path model with direct and indirect effects. T1, Time 1; T2, Time 2; T3, Time 3; WFC, work–family conflict; ACB, altruistic citizenship behavior; CWS, coworker support. **p < 0.01; *p < 0.05;^ NS^, not significant.

The hypothesized relationships were tested in a three-wave longitudinal autoregressive path model with direct and indirect effects (Figure 2). The significance of hypothesized relationships was tested by *t* values that were obtained by using bias-corrected bootstrapped confidence intervals method in SmartPLS, with 5000 samples. It is a two-unit time-lagged model that has been developed based on the longitudinal mediation estimation approach illustrated in Cole and Maxwell (2003) and Maxwell et al. (2011).

Our particular interest was in estimating the direct effect of time-1 ACB on time-2 CWS (Hypothesis 1), the effect of time-2 CWS on time-3 WFC (Hypothesis 2a), the direct effect of time-1 ACB on time-3 WFC (Hypothesis 2b), and the indirect effect of time-1 ACB on time-3 WFC through time-2 CWS (Hypothesis 2b).

The results of Path *a* indicate that ACB at time-1 (T1-ACB) is positively and significantly associated with CWS at time-2 (β = 0.34; *t*-value = 7.53; *p* < 0.001). This result supports our Hypothesis 1. The estimated Path *b* shows a negative and significant effect of time-2 CWS on time-3 WFC (β = −0.19; *t*-value = 3.46; *p* < 0.001) and supports our Hypothesis 2a.

Following Cole and Maxwell (2003), the overall direct effect of time-1 ACB on time-3 WFC was estimated on the paths that do not pass through the mediator. This effect was estimated along the paths represented by the dotted lines in Figure 2. According to Cole and Maxwell (2003), “the overall direct effect consists of the sum of all time-specific effects that start with X_1_ and end with Y_*T*_, but never pass through M” (p. 572). Following the direct effect estimation procedures explained in Cole and Maxwell (2003, pp. 576–577), the path coefficient for an overall direct effect was estimated as [β = (0.16 × 0.04) + (0.44 × −0.02) = −0.002]. The bootstrapping based on bias-corrected bootstrapped confidence intervals generated a *t*-value equal to 0.70. This value shows that the direct effect is not significant.

The overall indirect effect was measured on Paths *a* and *b* that represent the tracing where time-1 ACB (T1-ACB) affects time-3 WFC (T3-WFC) through time-2 CWS (T2-CWS). As a general rule, indirect effect is obtained by multiplying the coefficient Paths *a* and *b* (Cole and Maxwell, 2003; Hair et al., 2017). In our model, this effect was significant (β = −0.06; *t*-value = 3.12; *p* = 0.002). It supports out Hypothesis 2b.

## Discussion

This study used a longitudinal design to predict over time the effect of ACB on WFC. This study addressed the paradox of citizenship cost by hypothesizing an indirect rather than a direct effect of ACB on employee WFC through CWS. The findings of this study indicate that the relationship between altruistic behavior and WFC is a multivariate rather than a bivariate phenomenon where altruistic behavior affects employee WFC indirectly through CWS. The negative indirect association between altruistic behavior and WFC reflects that the costs of citizenship behavior are paradoxical.

Specifically, we drew upon the norms of reciprocity and equity theory to examine the relationship between ACB and CWS. As was predicted, the results indicate that ACB is positively associated with CWS. Path *a* in Figure 2 is positive and significant (β = 0.34; *t*-value = 7.53; *p* < 0.001). Similarly, Path *b* in Figure 2 is negative and significant (β = −0.19; *t*-value = 3.46; *p* < 0.001). It supports our Hypothesis 2a that CWS is negatively associated with WFC. The indirect effect, which is obtained by multiplying Paths *a* and *b*, is negative and significant (β = −0.06; *t*-value = 3.12; *p* = 0.002). It supports our Hypothesis 2b that there is a negative indirect effect of ACB on WFC through CWS.

Though the focus of our analysis was indirect effect, examining direct effect was relevant to understand that the indirect effect reflects a true causal relationship, while the direct effect is meaningless when the intervening mechanisms are held constant (MacKinnon et al., 2000; Shrout and Bolger, 2002). In other words, if CWS is held constant, ACB is likely to make no effect on WFC, as is shown by insignificant direct effect (dotted lines in Figure 2). It implies that a theoretically viable relationship may not be valid in the absence of an exposure to intervening mechanisms. Specifically, it can be stated that removing CWS from the link between altruistic behavior and WFC would reflect no effect. We analyzed our model without exposure to the intervening variable (CWS) and found that the longitudinal direct effect of altruistic behavior on WFC was insignificant (β = −0.002; *t*-value = 0.70; *p* > 0.05).

The arguments of dark side of OCB in terms of hypothesizing a positive association between citizenship behavior and WFC are compelling. However, in a sample of employees who receive CWS in response to engaging in altruistic behavior, these hypotheses do not keep up. Insights from norms of reciprocity suggest that altruism is a strong human trait, and people receive support from others for being altruistic. The support from others provides a natural tool for avoiding WFC.

As discussed earlier, this study found a significant negative indirect effect of altruistic behavior on WFC through CWS and a very small insignificant negative direct effect of altruistic behavior on WFC. The contribution of this study can be evaluated in light of existing research on direct and indirect effects of OCB on WFC, though we do not believe in a direct effect, specifically, in a longitudinal analysis. With respect to direct effect, the findings are not consistent with Bolino and Turnley (2005) and Halbesleben et al. (2009) because the direct relationship between ACB and WFC is negative and insignificant. The longitudinal nature of this study contributes to this line of research by indicating that the significance of direct effect depends much on testing this effect over time. Once the direct effect is insignificant, it can be concluded that there is little harm (i.e., WFC) for an employee who engages in altruistic behavior.

There are studies that have examined a direct negative effect of OCB on WFC. For example, Bragger et al. (2005) found a negative direct relationship between OCB and WFC. Similarly, Tziner and Sharoni (2014) examined both direct and indirect effects of OCB on WFC in a cross-sectional study and found a significant negative direct effect. In both studies, the arguments and data analyses are compelling, but the cross-sectional nature of these studies makes the results less reliable. Deery et al. (2017), however, conducted a time-lagged study and found a negative but insignificant direct effect of altruism on WFC. Consistent with this line of research, our study suggests that the effect of altruistic behavior on WFC is less likely until the mediating processes are considered over time.

With respect to indirect effect, previous research lacks evidence on longitudinal analyses where true effects are likely to be determined. Tziner and Sharoni’s (2014) cross-sectional study, however, found a negative indirect effect of OCB on WFC through employee stress. Their results indicate that an “increased OCB subsequently reduces stress. Stress directly impacts work-family conflict, so that when stress decreases, the respondents experience less work-family” (p.41).

The findings of our study are consistent with Lam et al.’s (2016), “enrichment-based perspective,” which suggests that rather than depleting, OCBs enrich employee resources in terms of feelings of energy and meaningfulness at work. This perspective states that “doing good for others or for the organization is not only beneficial for the team, but it can also be positive for the wellbeing of the individuals who engage in those behaviors” (Lam et al., 2016, p. 388). The findings of our study support the idea that altruistic behaviors benefit employees in terms of enhanced support from coworkers, which helps reduce WFC.

### Theoretical Implications

There is an ongoing debate on the positive and negative outcomes of employee citizenship behavior. The present study contributes to this debate by providing empirical evidence on beneficial effect of performing altruistic behavior. The dark side studies have ignored the indirect pathways that emerge over time and leave beneficial rather than harmful effects on employee outcomes. This study takes altruistic behavior as a resource-generating rather than a resource-depleting process when its effects are examined over time. The negative indirect effect of altruistic behavior on WFC shows that resource conservation perspective supersedes resource drain perspective in a way that resource depletion effect of altruistic behaviors is not sustainable when reciprocity and social support, over time, are integrated with these perspectives. Similarly, role conflict perspective may provide misleading insights when bivariate association between altruistic behavior and WFC is examined by ignoring that “supportive members of a person’s role set(s) may directly reduce certain role pressures” (Greenhaus and Beutell, 1985, p. 86) over time. The integration of role perspective with social support perspective over time will provide a stronger argument and true causal findings for the multivariate phenomenon of the relationship between altruistic behavior and WFC.

Using principles of reciprocity and equity theory, we make a point that “a burdensome situation of exhibiting altruism is less likely to occur” when the provider bears net costs. It suggests that the absence of reciprocity to these behaviors (in the form of social support) may put the existence of these behaviors in danger in organizational and social life, and there will be no altruism. It, however, is against the basic rule of “cooperative social existence” of human beings (Hoffman, 1978). Consistency in altruistic and social support behavior fits well in the idea of “survival of the fittest” and suggests that consistently performing such behaviors “at work can have positive effects for family life” (Aw et al., 2021, p. 61). It suggests that altruistic behaviors reduce WFC because these behaviors nurture in an environment of reciprocation and social support. Without considering reciprocity and social support over time, the analysis of OCB may leave flaws in understanding the prevalence of OCBs in work life.

For researchers and managers estimating the costs of altruistic behaviors to suggest preventive measures, neglecting the indirect effects (over time) of the relationship between altruistic behavior and WFC may lead to an important misunderstanding. For example, if altruistic behaviors are reciprocated in terms of coworker or organizational support, the possibility that an employee (who engages in such behaviors) will suffer WFC may be overestimated, when the researchers/managers focus only on direct effect at a specific time.

### Practical Implications

Employees who do not perform altruistic behaviors are more likely to experience higher WFC due to other factors (that enhance WFC) than those employees who exhibit OCBs and receive support from coworkers. In other words, the factors (other than OCB) that enhance WFC are more likely to leave adverse effects on those employees who do not perform OCBs when compared with the employees who receive CWS in response to exhibiting OCBs. For organizational managers, promoting a culture of CWS by encouraging altruistic behaviors can be a most viable strategy to reduce WFC among their employees.

Given that altruistic behaviors benefit employees, organizational managers need to look into the ways and practices that enhance such behaviors. A recent study has delineated that organizations’ use of corporate social responsibility (CSR) practices enhances OCB (Ahmad et al., 2020). Other ways to enhance altruistic behaviors may be supervisory communication (Chan and Kuok, 2020), leader humility (Tuan et al., 2021), ethical work climate and high-quality leader–member exchange (Teng et al., 2020), spiritual leadership (Djaelani et al., 2020), humble leadership (Ding et al., 2020), etc. Considering altruistic behaviors in performance appraisals may also help.

### Limitations and Future Research

In spite of its contribution to the literature in organizational psychology, this study is not free of limitations. The first limitation of this study is that the sample is from the banking sector of Pakistan, and due to this, the issue of external validity of our results may arise. However, this study succeeded in explaining a causal mechanism through which altruistic behavior exerts its effect on WFC. It is important because “causal explanation is an important route to the generalization of causal descriptions because it tells us which features of the causal relationship are essential to transfer to other situations” (Shadish et al., 2002, p. 10). Future researchers can replicate the findings of this study for “the generalizability of statistical results” and “may advance non-statistical argument to generalize findings from larger populations of interest” (Bonett, 2012, p. 409).

Second, insights from previous research suggest that true indirect effects should be interpreted in light of their boundary conditions (Hayes, 2018). For example, coworkers’ norms of reciprocity (Halbesleben and Wheeler, 2011) may determine the propensity to reciprocate positive behaviors. Consequently, it may affect the intensity of indirect relationship between altruistic behavior and WFC, through WFC. Similarly, job autonomy (Liu et al., 2017) can make a difference in exerting the indirect effect of altruistic behaviors because greater job autonomy will allow employees to adjust time and activities for engaging in OCBs and supporting coworkers, while lower job autonomy may act otherwise. In the same way, an employee’s personality has much to do with work–life conflict and can be used as a boundary condition for the indirect relationship tested in this research. For example, Wayne et al.’s (2004) study found that agreeableness and conscientiousness were negatively associated while neuroticism and openness to experience were positively associated with WFC. Future researchers can examine the true causality between altruistic behavior and WFC by considering employee personality traits, job autonomy, coworker norms of reciprocity, and other factors as its boundary conditions.

Third, this study did not test other possible illustrations of the research model. Yu et al.’s (2018) longitudinal study examined the effect of work–family interferences on OCB through job satisfaction and found a significant indirect relationship. Their study is interesting and warrants our findings to be tested in reverse causality. It might be interesting if future researchers examine whether the employees, who are faced with WFC due to any reason, engage in altruistic behaviors and obtain CWS.

## Conclusion

This study supports the bright side of the effects of citizenship behavior. Specifically, it concludes that altruistic behaviors are likely to encourage beneficial outcomes while discouraging negative consequences in an employee’s work life. Moreover, the indirect rather than direct effect over time represents the true effect of altruistic behavior on WFC.

## Data Availability Statement

The raw data supporting the conclusions of this article will be made available by the authors, without undue reservation.

## Ethics Statement

The studies involving human participants were reviewed and approved by the Ethical Committee for Scientific Research (ECSR) at COMSATS, Vehari. The patients/participants provided their written informed consent to participate in this study.

## Author Contributions

SH initiated the basic idea and wrote the main part of the manuscript. CD-P-H built the article structure. MD-P-H improved the manuscript. All authors contributed to the article and approved the submitted version.

## Conflict of Interest

The authors declare that the research was conducted in the absence of any commercial or financial relationships that could be construed as a potential conflict of interest.
